# Biotreatment of oily sludge by a bacterial consortium: Effect of bioprocess conditions on biodegradation efficiency and bacterial community structure

**DOI:** 10.3389/fmicb.2022.998076

**Published:** 2022-09-21

**Authors:** Dorra Hentati, Raeid M. M. Abed, Nasser Abotalib, Ashraf M. El Nayal, Ijaz Ashraf, Wael Ismail

**Affiliations:** ^1^Environmental Biotechnology Program, Department of Life Sciences, College of Graduate Studies, Arabian Gulf University, Manama, Bahrain; ^2^Department of Biology, College of Science, Sultan Qaboos University, Muscat, Oman; ^3^Bahrain Petroleum Company, Manama, Bahrain

**Keywords:** environmental pollution, hydrocarbons, biodegradation, petroleum, bacterial consortia, *Pseudomonas*

## Abstract

We studied the biodegradation of oily sludge generated by a petroleum plant in Bahrain by a bacterial consortium (termed as AK6) under different bioprocess conditions. Biodegradation of petroleum hydrocarbons in oily sludge (C_11_-C_29_) increased from 24% after two days to 99% after 9 days of incubation in cultures containing 5% (w/v) of oily sludge at 40°C. When the nitrogen source was excluded from the batch cultures, hydrocarbon biodegradation dropped to 45% within 7 days. The hydrocarbon biodegradation decreased also by increasing the salinity to 3% and the temperature above 40°C. AK6 tolerated up to 50% (w/v) oily sludge and degraded 60% of the dichloromethane-extractable oil fraction. Illumina-MiSeq analyses revealed that the AK6 consortium was mainly composed of *Gammaproteobacteria* (ca. 98% of total sequences), with most sequences belonging to *Klebsiella* (77.6% of total sequences), *Enterobacter* (16.7%) and *Salmonella* (5%). Prominent shifts in the bacterial composition of the consortium were observed when the temperature and initial sludge concentration increased, and the nitrogen source was excluded, favoring sequences belonging to *Pseudomonas* and *Stenotrophomonas*. The AK6 consortium is endowed with a strong oily sludge tolerance and biodegradation capability under different bioprocess conditions, where *Pseudomonas* spp. appear to be crucial for hydrocarbon biodegradation.

## Introduction

It has been recognized that anthropogenic activities throughout the fossil fuel industry are key contributors to environmental pollution (Imam et al., 2021). Worldwide, the petroleum industry generates massive quantities of waste oily sludge during oil production, transportation, storage and refining operations (Hu et al., 2013; Peng et al., 2022) which were estimated to be more than one billion tons of waste sludge annually (Roy et al., 2018). This quantity is anticipated to increase due to upsurge in oil production and refining to satisfy the rising global energy demand (Hu et al., 2013; Imam et al., 2021). Refining oily sludge is a recalcitrant residue and a complex mixture of oil/water emulsion, solid particles, organic compounds and heavy metals (Sarkar et al., 2020). Because it contains high concentrations of heavy, toxic, carcinogenic and mutagenic constituents (benzene, phenol, polycyclic aromatic hydrocarbons, resins, asphaltenes and heavy metals), refining oily sludge has been designated as a hazardous waste by the Resource Conservation and Recovery Act (RCRA) (US EPA, 2016). Management of this waste is a major challenge for the petroleum industry since improper handling may cause severe damage to the environment and public health (Varjani et al., 2020).

Various physicochemical methods such as pyrolysis, incineration, solvent extraction, centrifugation and ultrasonic irradiation have been developed for the treatment and disposal of refining oily sludge (Johnson and Affam, 2019). However, these methods suffer from many limitations such as high energy consumption, harsh operating conditions and high cost along with the generation of secondary pollution, making them non-ecofriendly (Jasmine and Mukherji, 2019; Ke et al., 2021). On the contrary, bioremediation has gained increasing interest as alternatives to the physicochemical treatments and an integral contributor to bio-economy and sustainable development (Wang et al., 2016; Ismail et al., 2017; Imron et al., 2020; Imam et al., 2021). There is a large number of studies on bioremediation of crude oil- and hydrocarbon-polluted environments (Hentati et al., 2016; Imron et al., 2020; Imam et al., 2021). However, such studies on waste oily sludge are still limited, probably due to its structural complexity and higher recalcitrance (Suganthi et al., 2018; Ke et al., 2021).

Bioremediation is highly governed by the type, abundance and biodegradation potential of the involved microorganisms (Varjani, 2017). It was shown that use of microbial consortia for disposal of complex pollutants, like refining oily sludge, could be more advantageous compared to individual microorganisms (Suganthi et al., 2018) due to synergistic interactions among different members of the consortium (Sarkar et al., 2020). Furthermore, pH, salinity, temperature, oxygen and nutrients availability, as well as nature and concentration of the pollutants are among the physicochemical factors that profoundly influence biodegradation rates (Imron et al., 2020). These environmental parameters could also influence the microbial community structure and function in polluted ecosystems (Liu et al., 2017; Alvarez et al., 2022). Therefore, both biotic and abiotic factors must be empirically optimized to develop effective bioremediation strategies for the disposal of petroleum hydrocarbon pollutants (Liu et al., 2017; Roy et al., 2018).

Some studies reported oily sludge biodegradation using microbial cultures (Jasmine and Mukherji, 2019; Varjani et al., 2020). However, it is currently not sufficiently understood how bioprocess conditions can influence refining oily sludge biotreatment and the structure of the involved microbial communities. Therefore, in this study we used a bacterial consortium to treat waste oily sludge collected from a petroleum plant in Bahrain in batch cultures. We studied the effect of various bioprocess conditions on the biodegradability of oily sludge hydrocarbons and the bacterial consortium composition.

## Materials and methods

### Oily sludge source and characterization

The refining oily sludge was collected on March 2020 from the weathering pit of a petroleum plant in Bahrain. Physicochemical analyses of the oily sludge were performed according to standard methods (Supplementary Table 1). Total petroleum hydrocarbons contained in the oily sludge were measured as described by Tahhan et al. (2011) with slight modification. Briefly, 6 g of oily sludge were air-dried to a constant weight and triplicate 1 g samples of the air-dried oily sludge were consecutively extracted with 100 mL each of hexane, dichloromethane, and chloroform. All three extracts were pooled and dried using a rotary evaporator. After solvent evaporation, the amount of recovered oil was determined gravimetrically. The extracts obtained were subsequently analyzed by Gas Chromatography-Mass Spectrometry (GC-MS) according to Jasmine and Mukherji (2015).

### Bacteria and culture media

The AK6 consortium was previously isolated from a hydrocarbon-polluted soil (from Kuwait) in chemically defined medium (CDM) containing dibenzothiophene as the sole sulfur source and glucose as a carbon source (Ismail et al., 2016). CDM was prepared from stock solutions as a basal medium (phosphate buffer, NH_4_Cl and deionized water) and supplemented with vitamins, trace elements, FeCl_2_, CaCl_2_, MgCl_2_ (these components are designated hereafter as the “complement”) (Supplementary Table 2). For the preparation of the starter culture, AK6 (1 mL), preserved at −80°C, was inoculated into a 100 mL Erlenmeyer flask containing 20 mL LB (Luria-Bertani) broth and incubated in an orbital shaker (GFL 3033, Germany) for 24 h at 30°C and 180 rpm. Then, 10 mL from the culture were transferred into a 1 L Erlenmeyer flask containing 400 mL LB broth and incubated in an orbital shaker at 30°C for 24 h. The cells were harvested by centrifugation (16,000 x g, 10 min) in a Sorvall Lynx 6000 centrifuge (ThermoScientific, Germany), and washed twice with phosphate buffer (0.1 M, pH 7). The washed cell pellet was re-suspended in 50 mL of phosphate buffer and this cell suspension was used to inoculate batch cultures (5%) to reach a biomass load about 12 g/L (dry cell weight).

### Biodegradation of oily sludge by the AK6 consortium

Sludge biodegradation was studied in batch cultures (50 mL) in CDM containing 5% (w/v) of oily sludge as the sole carbon and sulfur source and the cultures were incubated at 40°C in an orbital shaker (180 rpm), for 7 days. Cultures without bacterial inoculation were used as abiotic controls for all biodegradation experiments and treated under the same conditions. All experiments were performed in biological duplicates. Whole cultures and abiotic controls were extracted with an equal volume of dichloromethane at the end of the incubation period. The organic phase was then evaporated and the residual oil was analyzed by GC-MS on a Shimadzu gas chromatography system (GC 2010 plus) coupled with a Shimadzu MS–QP2020 mass detector with a capillary RXI-5SILMS column (length 30 m, internal diameter 0.25 mm, film thickness 0.4 μm, Restek, USA).

Helium was used as carrier gas with a flow rate of 3 mL/min, in a split ratio of 20. The temperature was set at 55°C and then increased to 300°C at 5°C/min rate and maintained at 300°C for 40 min. The obtained MS spectra were compared with the reference spectra present in the NIST.Lib (Spectral match factor of at least 90%).

The residual hydrocarbons were represented as the sum of the total peak areas of hydrocarbons detected by GC-MS and the percentage of hydrocarbons degradation was calculated using the following equation:


$$\left(\%\right)\mathrm{Degradation}=\frac{\begin{array}{c} \mathrm{Initial}\,\mathrm{total}\,\mathrm{area}\,\mathrm{of}\,\mathrm{hydrocarbons}-\mathrm{Final}\,\mathrm{total}\,\mathrm{area}\,\mathrm{of}\,\mathrm{hydrocarbons} \end{array}}{\mathrm{Initial}\,\mathrm{total}\,\mathrm{area}\,\mathrm{of}\,\mathrm{hydrocarbons}}\times 100$$

### Biodegradation of oily sludge under different bioprocess conditions

To investigate how culturing conditions affect the biodegradability of the refining oily sludge, we grew the AK6 consortium on sludge as the sole carbon and sulfur source in batch cultures under different conditions following a one-variable-at a time approach. Accordingly, in each experiment one condition was individually manipulated, while all other conditions were fixed. The studied factors included temperature (25, 30, 35, 40, 45, 50, and 55°C), salinity (0, 15, 30, and 50 g/L NaCl), pH (6, 7, and 8), shaking speed (120, 180, and 250 rpm), concentration of oily sludge (5%, 25%, and 50%, w/v), incubation time (0, 2, 4, 7, and 9 days) and culture medium composition (complete CDM, CDM without vitamins, CDM without trace elements, CDM without MgCl_2_, CDM without CaCl_2_, CDM without FeCl_2_, CDM without NH_4_Cl (nitrogen source), and basal CDM (consists only of phosphate buffer, NH_4_Cl and deionized water)). Furthermore, three different inoculum sizes (biomass load): 12, 27 and 7.3 g/L (dry weight), were evaluated after 2 and 7 days of incubation. All experiments were performed in biological duplicates. The oily sludge was extracted from all cultures and abiotic controls and analyzed as described earlier.

### Analysis of community structure under different bioprocess conditions

Bacterial genomic DNA was isolated from the sludge biodegradation cultures using BioVision kit (Biovision Inc., CA, USA), quantification of DNA was performed using a Qubit fluorometer (Invitrogen, Thermo Fisher Scientific, Singapore) and the DNA purity was checked using a DS-11 FX^+^ spectrophotometer/Fluorometer (DeNovix Inc., USA). Purified DNA extracts were submitted to Genomics BioSci & Tech Co., Ltd. (New Taipei City, Taiwan) for paired-end Illumina- MiSeq sequencing of the V3-V4 hypervariable region of the bacterial 16S rRNA gene as described (AlDhafiri et al., 2022) using the forward primer: CCTACGGGNGGCWGCAG and reverse primer: GACTACNVGGGTATCTAATCC (Klindworth et al., 2013) (see Supplementary Material for details). Sequences were generated on two separate MiSeq runs using a single indexing strategy. Samples were demultiplexed and sequencing adapters, barcodes, and primers were removed using the *fastqprocessor* developed by MRDNA (Shallowater, TX, USA), which further ensured the correct orientation of all reads as forward (R1) and reverse (R2). After demultiplexing, intact read pairs were extracted using *pairfq_lite^1^* . Further processing was conducted in R v3.5.2 (R Core Team, 2018) using the R package dada2 v1.10.1 (Callahan et al., 2016) with default parameters if not otherwise indicated. Quality filtering was conducted at a maximum expected error rate of 3 for both forward and reverse reads after reads were truncated to 230 bp. Error learning (based on at least 10^8^ bases) and denoising (pooling all sequences per run) were executed separately for each MiSeq run. Afterward, forward and reverse reads of each sample were merged with a minimum overlap of 10 bp, and chimera detection was performed with the method ‘consensus’ on the whole dataset. Furthermore, only sequences between 400 and 430 bp as well as those occurring at least twice in the data set were retained. Taxonomic classification was conducted using the SILVA NGS web-service with the SILVA ribosomal database (version 138) (Quast et al., 2012). Only bacterial sequences classified on phylum level with a sequence similarity of at least 93% to the reference database and not affiliated with chloroplasts and mitochondria were used for the further analysis. Operational taxonomic units (OTUs) were defined as unique amplicon sequence variants. OTU richness, Chao1 index, Inverse Simpson Index, the absolute number of singletons (SSO_*abs*_) and doubletons (DSO_*abs*_) were calculated using R-Studio, version 1.0.153 (R Core Team, 2018) using the same number of sequences for all datasets (67,787 sequences). Cluster analysis Bray-Curtis dissimilarities were calculated based on relative sequence abundances of OTUs without further transformation in order to visualize shifts in the bacterial community composition. The dataset was submitted to Sequence Read Archives of the GenBank under accession numbers SRR17054280-SRR17054310 (BioProject PRJNA739293).

### Statistical analysis

All values represent the mean ± standard deviation. Data were statistically analyzed by one-way ANOVA using Tukey’s multiple comparisons test and applying a significance level of *p* < 0.05, using GraphPad Prism 6 (Trial version).

## Results

### Characterization and biodegradation of the oily sludge by the bacterial consortium AK6

The oily sludge was slightly acidic (pH 5.6), with a water content of 49.63% and a viscosity of 273.3 Cst (Centistock, mm^2^/s) (Supplementary Table 1). A high content of sulfur (14 g/kg) was recorded, while nitrogen and phosphate contents were relatively lower (681 and 149 mg/kg, respectively). Iron, silicon and aluminum were detected at considerably higher concentrations (4462, 2062, and 1019 mg/kg, respectively), while zinc, lead, copper, manganese, vanadium and nickel were present at relatively lower concentrations (between 21-202 mg/kg). Total petroleum hydrocarbon content in the oily sludge was around 53 ± 2.5%. Analysis by GC-MS showed the presence of various hydrocarbons including essentially *n*-alkanes ranging from C_11_ to C_32_, as well as the individual isoprenoid hydrocarbons, pristane and phytane (Supplementary Figure 1 and Supplementary Table 3).

**FIGURE 1:**
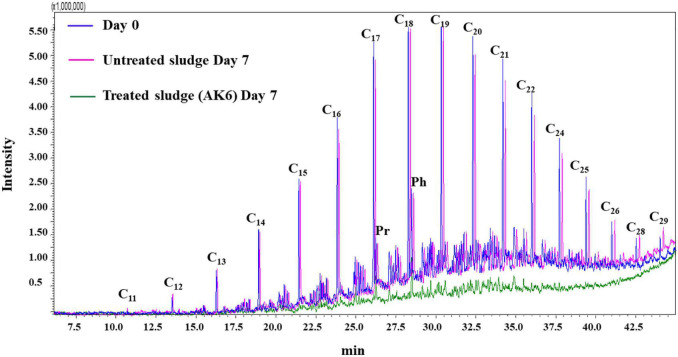
Total ion chromatograms showing biodegradation of oily sludge hydrocarbons (extracted in dichloromethane) by the mixed culture AK6. The batch cultures contained oily sludge (5%, w/v) as the sole carbon and sulfur source and were incubated for 7 days at 40°C under shaking at 180 rpm. (Pr): pristane; (Ph): phytane.

GC-MS analyses revealed a high capacity of the AK6 consortium to break down petroleum hydrocarbons (C_11_-C_29_ alkanes) of the oily sludge (Figure 1). The AK6 bacterial consortium was able to metabolize about 90 ± 5.2% of dichloromethane-extractable hydrocarbons contained in 5% (w/v) of the oily sludge within 7 days of incubation at 40°C under shaking at 180 rpm.

### Biological treatment of the oily sludge under different bioprocess conditions

**FIGURE 2:**
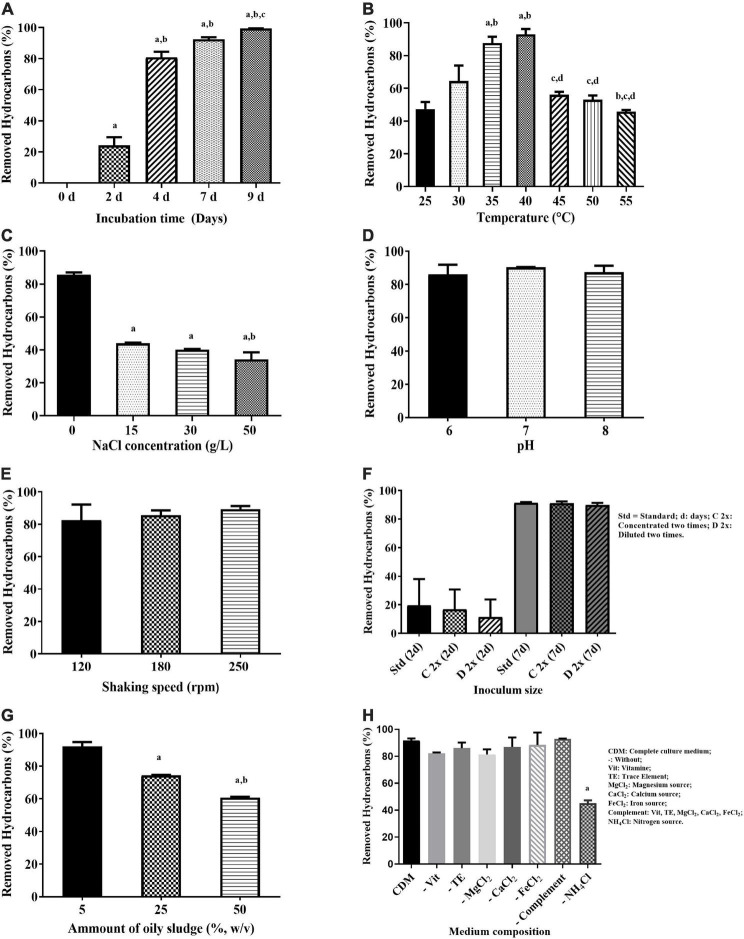
Evaluation of the effect of incubation time **(A)**, temperature **(B)**, NaCl concentration **(C)**, pH **(D)**, shaking speed **(E)**, inoculum size **(F)** amount of oily sludge **(G)**, and culture medium composition **(H)** on the biodegradation of oily sludge by mixed culture AK6. Values given represent the mean of two replicates ± standard deviation. vs: versus. **(A)** **^a^** *p* < 0.05 at 0 day vs. other incubation times; **^b^** *p* < 0.05 at 2 days vs. other incubation times; **^c^** *p* < 0.05 at 4 days vs. other incubation times; **(B)** **^a^** *p* < 0.05 at 25°C vs. other temperatures; **^b^** *p* < 0.05 at 30°C vs. other temperatures; **^c^** *p* < 0.05 at 35°C vs. other temperatures; **^d^** *p* < 0.05 at 40°C vs. other temperatures; **(C)** **^a^** *p* < 0.05 at 0 g/L NaCl vs. other salinities; **^b^** *p* < 0.05 at 15 g/L NaCl vs. other salinities; **(G)** **^a^** *p* < 0.05 at 5% oily sludge vs. other oily sludge concentrations; **^b^** *p* < 0.05 at 25% oily sludge vs. other oily sludge concentrations; **(H)** **^a^** *p* < 0.05 complete culture medium vs. medium without complement (vitamins, trace elements, MgCl_2_, CaCl_2_ and FeCl_2_) and medium without NH_4_Cl (the nitrogen source). The pH, shaking speed and inoculum size had no significant influence (*p* > 0.05) on hydrocarbon removal by AK6.

As illustrated in Figure 2A, the net hydrocarbon loss increased with the incubation time, reaching almost 100% after 9 days. The most remarkable increase occurred between the second and fourth day, followed by slower hydrocarbon removal up to day 9. Furthermore, biodegradation of oily sludge hydrocarbons increased by rising the temperature, with the highest hydrocarbon removal of 87.6 ± 3.8 and 92.7 ± 3.3% attained at 35 and 40°C, respectively (Figure 2B). The degradation activity significantly decreased (56.1 ± 1.7%) (*p* < 0.05) by increasing the incubation temperature from 40°C to 45°C, followed by marginal decline up to 55°C.

Salinity was also found to have a remarkable effect on hydrocarbon biodegradation where it dropped by increasing the salinity of the culture medium. As shown in Figure 2C, hydrocarbon degradation was highest in the absence of NaCl then dropped by ∼ 50% as the NaCl concentration increased up to 50 g/L (*p* < 0.05). Unlike temperature and salinity, pH, shaking speed and inoculum size had no significant influence (*p* > 0.05) on the biodegradation of oily sludge hydrocarbons by the AK6 consortium (Figures 2D–F). In contrast, the degradation activity decreased as the sludge concentration increased in the culture medium (Figure 2G). The highest biodegradation activity of 92 ± 2.7% was attained in the presence of 5% (w/v) of oily sludge, whereas in the presence of 25 and 50% oily sludge, AK6 was able to degrade about 74.3 ± 0.3 and 60.7 ± 0.5% of sludge hydrocarbons, respectively.

Unexpectedly, AK6 maintained a high capacity to degrade oily sludge hydrocarbons (more than 80%) (*p* > 0.05) (Figure 2H) in the absence of essential nutrients (vitamins, trace elements, MgCl_2_, CaCl_2_, and FeCl_2_) in the culture medium either individually or altogether. Even when AK6 was grown on oily sludge in just basal medium (phosphate buffer, NH_4_Cl and deionized water), the hydrocarbon degradation activity (between 81 and 93%) was similar to that attained in complete CDM containing all nutrients. On the contrary, in CDM which lacks only the nitrogen source (NH_4_Cl) we noticed that hydrocarbon biodegradation by AK6 dropped almost to half (45.1 ± 2.1%) (*p* < 0.05) compared to cultures grown in complete CDM (Figure 2H). Biodegradation of oily sludge hydrocarbons by the AK6 consortium under different bioprocess conditions was also presented in terms of residual hydrocarbons as shown in Supplementary Figure 2.

### Composition and population dynamics of the AK6 consortium

#### Development of the AK6 bacterial composition as a function of sludge biotreatment time

**FIGURE 3:**
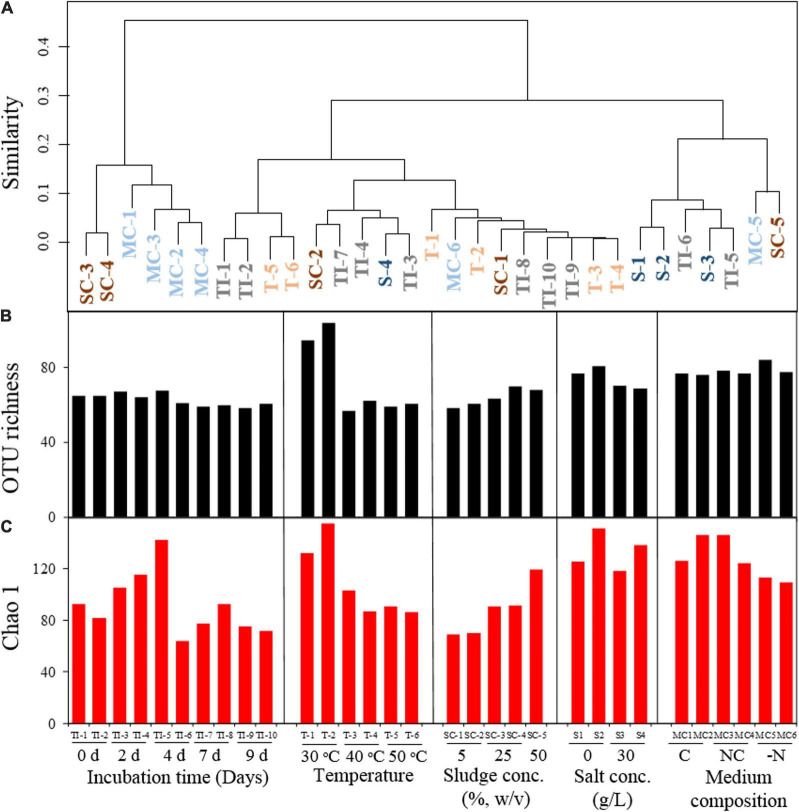
Hierarchical cluster diagram based on Bray-Curtis dissimilarity **(A)** comparing the AK6 bacterial culture composition under different oily sludge biotreatment conditions and a bar plot comparing the changes in OUT richness **(B)** and Chao1 diversity indices **(C)** among the different treatments. MC: medium composition [C: complete medium, NC: without complement (vitamins, trace elements, MgCl_2_, CaCl_2_ and FeCl_2_), -N: without nitrogen source].

**FIGURE 4:**
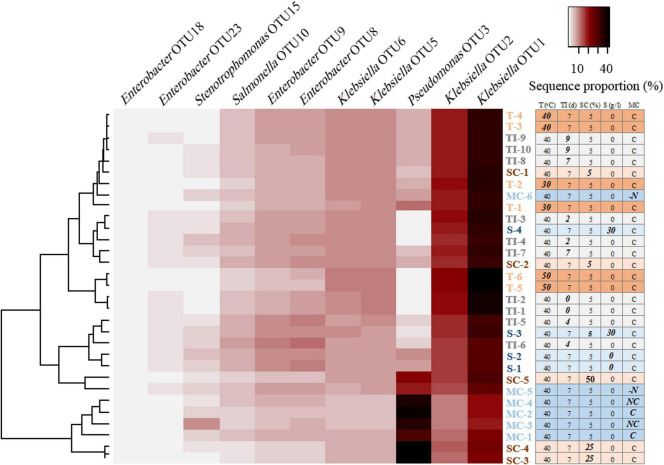
A heatmap representing comparing the relative abundance (% of total sequences) of the most abundant OTUs in the AK6 bacterial culture under different conditions of oily sludge biotreatment, indicated by the table. T: temperature, TI: incubation time, SC: sludge concentration (%, w/v), S: salinity (g/L), MC: medium composition [C: complete medium, NC: without complement (vitamins, trace elements, MgCl_2_, CaCl_2_ and FeCl_2_), -N: without nitrogen source].

**FIGURE 5:**
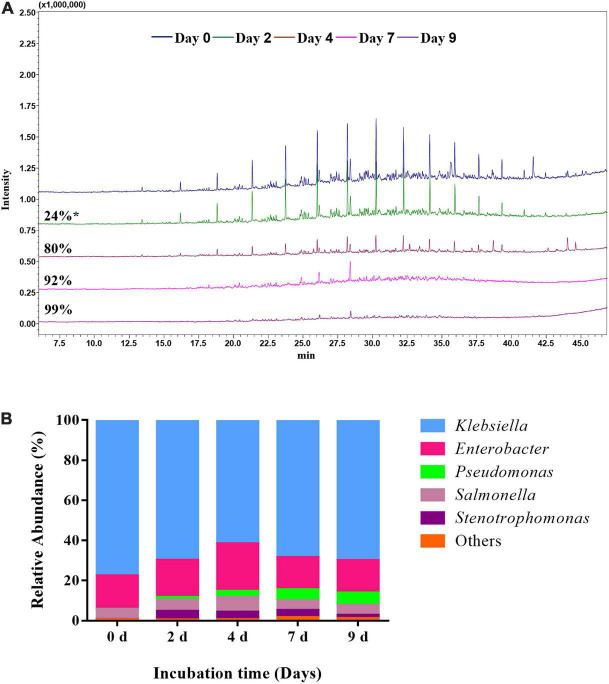
Total ion chromatograms **(A)** showing biodegradation of oily sludge hydrocarbons by the mixed culture AK6 at different time intervals (0, 2, 4, 7, and 9 days) and the associated compositional shifts in the structure of the AK6 consortium **(B)**. *: Hydrocarbon degradation rate.

Illumina-MiSeq amplicon sequencing revealed the bacterial composition of the AK6 consortium during treatment of the oily sludge at time intervals (0, 2, 4, 7, and 9 days). The majority of the sequences (≥ 98.2%) detected in AK6 belonged to *Gammaproteobacteria* and a small fraction of sequences (< 1.3%) was affiliated to *Alphaproteobacteria*. Cluster analysis of OTUs based on Bray-Curtis dissimilarities showed a clear shift in the bacterial community composition with the incubation time, although the OTU richness did not exhibit a drastic change over time (Figure 3). While the number of OTUs at the different incubation times ranged between 58 to 68 OTUs, the Chao 1 index ranged between 64 to 116, with slight differences at the different incubation times (Figure 3). At day 0, the original consortium was dominated by sequences belonging to the genera *Klebsiella* (77.6% of total sequences), *Enterobacter* (16.7%) and *Salmonella* (5%). After 2 days of incubation, the community composition remained more or less similar, however, after 4 days, the relative abundance declined for *Klebsiella* to 61% of total sequences, but increased for *Enterobacter* to 23% and this community shift coincided with the most remarkable increase in hydrocarbon biodegradation (Figures 4, 5). Sequences belonging to *Pseudomonas* and *Stenotrophomonas* were detectable in the culture only after 4 days of incubation onwards, exhibiting their highest relative abundance at 4-6% of total sequences at day 7 (Figures 4, 5). In the final consortium after 9 days of incubation, 70% of total sequences belonged to *Klebsiella*, 16% to *Enterobacter*, 6% to *Pseudomonas*, and 4.8% to *Salmonella* (Figures 4, 5).

#### Impact of temperature, salinity, oily sludge concentration, and medium composition on the AK6 bacterial community structure

**FIGURE 6:**
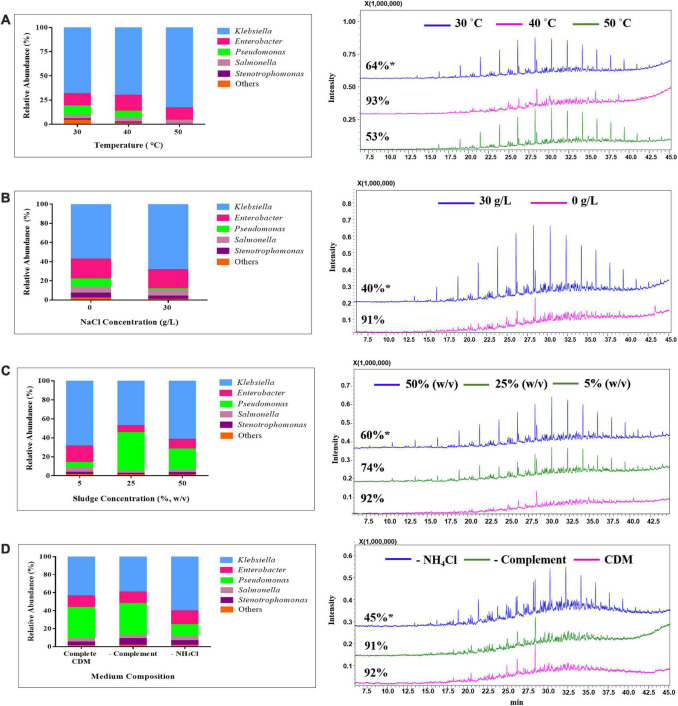
Biodegradation of oily sludge hydrocarbons and impact of temperature **(A)**, NaCl concentrations **(B)**, sludge concentration **(C)** and medium composition **(D)**, on the bacterial composition of the consortium AK6 at genus level. *: % hydrocarbon degradation.

The bacterial structure of the AK6 consortium shifted when incubated at different temperatures, as inferred from cluster analysis (Figure 3A), with the most significant shift occurring at 50°C. The diversity indices (i.e., OTU richness and Chao 1) were significantly higher at 30°C compared to 40 and 50°C. For instance, the number of OTUs in the duplicate samples at 30°C was 94 and 104, whereas it was below 57 in all replicates at 40 and 50°C. At 30°C, the consortium was mainly dominated by *Klebsiella* (68% of total sequences), *Enterobacter* (12.5%) and *Pseudomonas* (6.5%) (Figure 4). At 40°C, the bacterial communities in the duplicate samples fell into the same cluster with the duplicate samples at 30°C, with minor changes in the proportion of the major sequences. At 50°C, the proportion of *Klebsiella* increased to 83% of total sequences, whereas the proportion of *Pseudomonas* decreased to less than 0.1% and this was accompanied by a significant drop in hydrocarbon biodegradation (Figures 4, 6A).

The effect of salinity on the consortium composition was not very profound, except for a slight increase in the proportion of *Klebsiella* from 57% to 68% and a slight decrease in the proportion of *Pseudomonas* from 8.5% to 1.5% at 0 g/L and 30 g/L salt concentration, respectively, which occurred as the hydrocarbon removal declined (Figures 4, 6B). The relative abundance of the remaining members of the consortium remained more or less unchanged.

Cluster analysis showed that the use of different sludge concentrations has induced shifts in the structure of the bacterial consortium AK6 (Figure 3). At 5% sludge concentration, where hydrocarbon removal was the highest, *Klebsiella* and *Enterobacter* constituted the most dominant members of the community, with relative abundances of 68 and 17% of total sequences, respectively (Figures 4, 6C). However, the relative abundances of these bacteria decreased to 47 and 7% of total sequences, respectively when sludge concentration increased to 25%, with a strong upshift in the proportion of sequences belonging to *Pseudomonas* from 5 to 41% of total sequences was observed where the hydrocarbon degradation declined (Figures 4, 6C). At 50% sludge concentration, the consortium still maintained a high proportion of *Pseudomonas* sequences at 22% of total sequences, in addition to *Klebsiella* (65%) and *Enterobacter* (10%) (Figures 4, 6C).

Biotreatment of the oily sludge with the AK6 consortium in a complete medium and in a medium lacking nutrient (containing only phosphate and nitrogen source) resulted in the enrichment of the consortium members at comparable proportions (Figure 4). The consortium in these two treatments contained predominantly *Klebsiella*, *Pseudomonas* and *Enterobacter* at the average proportions of 41.5%, 34.8% and 12.8% of total sequences, respectively, a result reflecting the similar degradation rates under these two conditions (Figure 4). When the sludge biotreatment was performed in the absence of the nitrogen source (ammonium chloride) in the culture medium, the relative abundance of *Klebsiella* increased to 60.4%, whereas the relative abundance of *Pseudomonas* decreased to 13.2% of total sequences, which was accompanied by 50% decline in hydrocarbon removal (Figures 4, 6D).

## Discussion

GC-MS and AK6 community structure analysis confirmed biodegradation of oily sludge hydrocarbons, which is in line with the fact that the AK6 consortium was originally obtained from hydrocarbon-polluted soil (Ismail et al., 2016). Different species of the dominant genera (*Klebsiella*, *Pseudomonas* and *Stenotrophomonas*) in the oily sludge cultures of AK6 were frequently reported for their hydrocarbons, crude oil, and oily sludge biodegradation capabilities, and many of them were isolated from hydrocarbon-polluted environments (Chebbi et al., 2017; Behera et al., 2020; Bilen Ozyurek and Seyis Bilkay, 2020; Koolivand et al., 2020; Medić et al., 2020; Hentati et al., 2021a; Muneeswari et al., 2021; Choudhury et al., 2022). Furthermore, since no exogenous sulfur source was added to the batch cultures and chemical analysis revealed the presence of sulfur in the oily sludge, it can be hypothesized that AK6 relied on the oily sludge as the sulfur source, which is corroborated by the reported ability of AK6 to utilize different thiophenic organosulfur compounds commonly found in crude oil, as the sole sulfur source (Ismail et al., 2016). The % of hydrocarbon biodegradation by AK6 came within the range reported in the literature (Table 1). However, AK6 removed a higher % of hydrocarbons (90%) within a shorter time of incubation. It is, nonetheless, worth noting that comparing the results of different studies is not always straightforward and conclusive due to differences in the adopted experimental approaches and used materials. For instance, the studies listed in Table 1 reported different inoculum size/biomass load for the oily sludge biodegradation experiments, which could influence the hydrocarbon biodegradation rates or net hydrocarbon loss.

**TABLE 1:** Comparison of the oily sludge biodegradation by the bacterial consortium AK6 with other oily sludge-degrading consortia.

Microbial consortia composition	Source of microbial consortia	Initial biomass concentration	Initial concentration of oily sludge (%)	Oily sludge degradation (%, aliphatic fraction)	Incubation time (days)	Temperature and shaking speed	Reference
*Klebsiella, Enterobacter*, *Pseudomonas, Stenotrophomonas Salmonella* spp.	Oily sludge, Bahrain	12 g/L (dry weight)	5%	90 ± 5.2	7	40°C, 180 rpm	This study
*Shewanalla chilikensis* *Bacillus firmus* *Halomonas hamiltonii*	Petroleum oily sludge/oily sludge- contaminated soil, India	not reported	1%	97	21	37°C, 120 rpm	Suganthi et al., 2018
*Bacillus subtilis* *Bacillus megaterium* *P. fluorescens* *Candida tropicalis* *Rhodotorula dairenensis*	Petroleum-contaminated soil, China	5 × 10^6^CFU/mL	1%	94.3	30	30°C, 100 rpm	He et al., 2014
*Stenotrophomonas acidaminiphila* *Bacillus megaterium* *Bacillus cibi* *Pseudomonas aeruginosa* *Bacillus cereus*	Petrochemical oily sludge/ Petrochemical waste disposal soil, Brazil	10^5^ to 10^6^	1%	90.7	40	30°C, 100 rpm	Cerqueira et al., 2011
*Acinetobacter calcoaceticus* *Nocardiodes simplex* *P. alcaligenes* *Rhodotorula graminis*	Hydrocarbons-polluted soil/oily sludge waste, Spain	10^7^ cells/mL for Bacteria 10^6^ cells/mL for Yeasts	4%	100	10	30°C, 250 rpm	Gallego et al., 2007

The observed changes in the oily sludge hydrocarbon degradation and composition of the AK6 consortium under different conditions reflect known differences amongst hydrocarbon-degrading bacteria in terms of substrate range/preferences, growth rate and tolerance of hydrocarbon toxicity (Ismail et al., 2013; Minai-Tehrani et al., 2015; Xu et al., 2018; You et al., 2018). The increase in hydrocarbon degradation with time up to day 4 could be due to acclimatization of the AK6 community members, while the subsequent drop might reflect depletion of essential nutrients and/or readily biodegradable components and accumulation of toxic or less biodegradable metabolites/hydrocarbons (Minai-Tehrani et al., 2015; Xu et al., 2018; You et al., 2018). Higher relative abundance of *Enterobacter*, *Pseudomonas* and *Stenotrophomonas* that accompanied increase in the oily sludge biodegradation within 4 days suggests that members of these genera are key hydrocarbon degraders in the AK6 consortium. In view of the relatively initial lower abundance of *Pseudomonas* and *Stenotrophomonas* spp. compared to other AK6 members, this assumption might appear contradictory to the general belief assigning most abundant community members as the most influential in pollutant degradation process. However, this does not have to be always the case and some studies reported a crucial role of the so-called “rare biosphere” or “conditionally rare taxa” in pollutants biodegradation (Wang et al., 2017, 2021; Xu et al., 2018; Pascoal et al., 2020).

Recently, Muneeswari et al. (2021) reported the presence of genes related to petroleum hydrocarbons degradation in the genome of a hydrocarbonoclastic and biosurfactant-producing strain STP-3 of *Enterobacter xiangfangensis*. In addition, Sarkar et al. (2017) detected alkane hydroxylase (*alkB*) gene, the key enzyme of microbial alkane degradation, in a consortium consisting of different hydrocarbonoclastic genera including *Enterobacter*. *Pseudomonas* spp. have been widely described as efficient hydrocarbon degraders (Medić et al., 2020; Choudhury et al., 2022). Studies on numerous *Pseudomonas* spp. revealed the presence of various genes encoding enzymes involved in hydrocarbon degradation such as oxygenases, dehydrogenases, hydroxylases as well as different transporters and related transcriptional regulators (He et al., 2018; Das et al., 2020). Similarly, hydrocarbon degradation by numerous *Stenotrophomonas* strains have been reported (Behera et al., 2020; Larik et al., 2019). Kumari et al. (2017) isolated a *Stenotrophomonas* sp. IITR87 strain having the potential to utilize phenanthrene, pyrene and benzo(a)-pyrene as a sole carbon source and detected *nid* genes, which are involved in polycyclic aromatic hydrocarbon degradation.

The observed changes in the oily sludge biodegradability with the temperature can be attributed to alterations in the physicochemical properties of the oil, structure and function of the microbial communities and the rate of hydrocarbons metabolism by microorganisms (Abed et al., 2015). Ke et al. (2021) reported that increasing the temperature from 10 up to 40°C improved oily sludge biodegradation by a bacterial consortium. At 50°C, sludge biodegradation started to decrease and the optimal temperature was around 30-40°C. In addition, Ibrahim (2016) found that the degradation rate of used engine oil increased as the temperature increased from 25 to 37°C and from 45°C upwards there was a decline in the degradation process, which generally agrees with our findings. It appears that the higher hydrocarbon degradation activity that took place by increasing the temperature from 30 to 40°C was not due to changes in the AK6 consortium structure. It could be attributed to better biocatalytic activity of the involved enzymes and/or improved bioavailability of the oily sludge hydrocarbons (Abed et al., 2015; Imron et al., 2020). On the contrary, the decrease in hydrocarbon degradation at 50°C might be due to the observed downshift in the relative abundance of *Pseudomonas* spp., which speaks for a key role in the sludge biodegradation process. Nonetheless, improper functioning of the cell membrane and hydrocarbon degradation enzymes, as well as potential development of anoxia, at this high temperature can’t be excluded (Leahy and Colwell, 1990; Abed et al., 2006, 2015; Al-Hawash et al., 2018). Generally, occurrence of the highest hydrocarbon degradation between 35 and 40°C and the subsequent drop at higher temperatures might reflect the mesophilic nature of the dominant AK6 members.

The solubility of hydrocarbons decreases with increasing salt concentration (Truskewycz et al., 2019; Hentati et al., 2021b), thus limiting the bioavailability of hydrocarbons to microorganisms (Abed et al., 2006, 2015; Fathepure, 2014). These previous findings might explain why hydrocarbon removal by AK6 dropped to almost half as the salt concentration increased from 0 to 3% (Abed et al., 2015; Camacho-Montealegre et al., 2021; Li et al., 2022). In addition, high salt concentrations are detrimental to microbial life due to perturbation of the cell membrane integrity, poor oxygen solubility, denaturation of proteins as well as desiccation (Abed et al., 2006; Hentati et al., 2021a). Since the AK6 consortium exhibited only marginal compositional shifts by increasing the culture salinity, it appears that the change of the structure of the AK6 consortium was not the main trigger for the observed drop of hydrocarbon removal. The known salt tolerance of some strains of *Klebsiella*, *Enterobacter*, *Pseudomonas* and *Stenotrophomonas* (Roder et al., 2005; Sarkar et al., 2018; Elabed et al., 2019; Emadi et al., 2022) backs this assumption.

The observed decease in hydrocarbon biodegradation efficiency at higher sludge concentrations in the AK6 batch cultures agrees with previous studies (Cui et al., 2020) and may be due to accumulation of toxic compounds and metabolites in the culture medium (Lin et al., 2010) and/or oxygen and nutrient limitation (Leahy and Colwell, 1990; Ismail et al., 2013). The impact of the initial sludge concentration on the AK6 community structure revealed several take-home messages. First, the initial sludge concentration is a key determinant of the involved community composition (Cui et al., 2020; Gao et al., 2022). Second, the AK6 community shifts occurred exclusively in the most abundant members. Third, *Pseudomonas* spp. appear to be better adapted to higher hydrocarbon concentrations (Singh and Tiwary, 2017; Ray et al., 2021).

One of the interesting findings of our study is the ability of the AK6 consortium to maintain its sludge biodegradation capacity in basal medium (phosphate buffer, NH_4_Cl and water) lacking most of the essential nutritional supplements. It is, therefore, tempting to propose that the AK6 consortium benefited from some sludge components such as sulfur, Ca, Mg, K, Fe, Mn, Ni, and Cu. However, AK6 was probably not able to retrieve its full nitrogen requirements from the oily sludge. This assumption is based on the observed drop in the% hydrocarbon loss in cultures lacking NH_4_Cl.

Wei et al. (2020) reported that addition of a nitrogen source enhanced oil degradation by promoting the abundance of hydrocarbon degraders in crude oil-contaminated wetland soils. This explains why hydrocarbon biodegradation by AK6 decreased by 50% when the nitrogen source was excluded from the batch cultures. The key role of *Pseudomonas* spp. in the oily sludge hydrocarbon biodegradation can be further reconciled from the community structure analysis showing that the decline in hydrocarbon degradation in the NH_4_Cl-free cultures was accompanied by a strong downshift in *Pseudomonas* relative abundance. Furthermore, the hydrocarbon removal dropped despite the increase in the relative abundance of *Klebsiella*. Nonetheless, we do not exclude that members of both genera were actively involved in the biodegradation process because utilization of fuel-borne organonitrogen compounds as a nitrogen source was reported for *Pseudomonas* and *Klebsiella* spp. (Li et al., 2006; Morales and Le Borgne, 2014). Different species of *Klebsiella* have been isolated from various oil-contaminated areas and were shown to play an active role in petroleum biodegradation and harbor genes of aromatic compounds degradation (Rodrigues et al., 2009; Zafra et al., 2016; Rajkumari et al., 2018).

Our finding that the AK6 consortium maintained its sludge biodegradation capabilities over the pH range 6-8 is in line with the fact that most heterotrophic bacteria favor pH levels ranging from 6 to 8 (Imron et al., 2020). Furthermore, Sarkar et al., 2020 reported that hydrocarbons biodegradation is optimal in the pH range 6.5 - 8.5.

We expected to see higher hydrocarbon removal rates by increasing the shaking speed from 120 to 250 rpm (Srinivasarao Naik et al., 2011; Ibrahim, 2016), which was not the case. We assume that already at 120 rpm the AK6 batch cultures were oxygen-saturated. Ferreira et al. (2012) reported that once the maximum oxygen saturation level is reached, further augmentation of the agitation speed does not enhance the degradation rate because the aeration rate will not change. We should also consider the possibility that oily sludge biodegradation under shaking at 120 rpm already reached a maximum due to exhaustion of the degradable fraction.

Probably, the differences in biomass load that we applied were not large enough to bring about remarkable changes in the biodegradation efficiency. In addition, larger inoculum size could lead to faster depletion of oxygen and nutrients in the growth medium, which can prohibit further degradation (Ibrahim, 2016). Our assumptions for the effect of the shaking speed can be extrapolated to the inoculum size.

As we pointed out in the introduction, the development and implementation of effective bioremediation approaches for reclamation of oily sludge-polluted environments require in-depth understanding of the composition of the involved microbial communities as well as their functional and structural resilience under different environmental conditions (Liu et al., 2017), which is currently lacking (Truskewycz et al., 2019). This is particularly important in view of climate change, which can affect both the recalcitrance of the oily sludge (weathering effects) as well as the dynamics, abundance and functioning of the hydrocarbon-degrading microbial communities (Keitt et al., 2016; Naylor et al., 2020). In this context, our findings fill some knowledge gaps by: (i) revealing how the removal of hydrocarbons from oily sludge is affected by key physicochemical factors, (ii) highlighting the microbiological responses of the studied bacterial consortium under different oily sludge biotreatment conditions, and (iii) identifying potential key hydrocarbon degraders amongst the members of the studied consortium. These findings pave the way toward further investigations on large-scale bioprocesses focusing on deeper analysis of the biotreated oily sludge, as well as the function and robustness of the involved bacterial consortia.

## Conclusion

The AK6 consortium exhibited a good potential as an oily sludge-biodegrading bacterial mixed culture. Furthermore, AK6 remodeled its bacterial composition as a response to the bioprocess conditions, particularly, the incubation time, temperature, salinity, and the lack of nitrogen source, which profoundly affected the oily sludge biodegradation efficiency. Despite the relatively low abundance of *Pseudomonas* spp. in the AK6 consortium, they appear to be the most crucial for oily sludge biodegradation. Follow-up studies should adopt deeper analysis, for instance via two-dimensional gas chromatography (GC x GC), to uncover the degradability of various sludge hydrocarbon fractions. In addition, large-scale sludge biotreatment with AK6 in mesocosms is worth investigating.

## Data availability statement

The datasets presented in this study can be found in online repositories. The names of the repository/repositories and accession number(s) can be found below: https://www.ncbi.nlm.nih.gov/genbank/, BioProject PRJNA739293.

## Author contributions

DH contributed to experimental design, performed experiments, collected and analyzed data, and wrote the original draft. RA analyzed the Illumina-MiSeq data. NA performed GC-MS analysis. AEN helped with the PCR experiments. IA contributed resources. WI conceptualized the study, acquired fund, and managed the project. All authors reviewed and approved the final version of the manuscript.

## Footnotes

1
https://github.com/sestaton/Pairfq
