# Supplementary material for: Biotreatment of oily sludge by a bacterial consortium: Effect of bioprocess conditions on biodegradation efficiency and bacterial community structure
Source: Front Microbiol. 2022 Sep 21;13:998076. doi: 10.3389/fmicb.2022.998076 (PMC9532598; doi:10.3389/fmicb.2022.998076)
Supplement: Supplementary file 1 [file Data_Sheet_1.pdf]

Supplementary Material

Biotreatment of Oily Sludge by a Bacterial Consortium: Effect of Bioprocess Conditions on Biodegradation Efficiency and Bacterial Community Structure

Dorra Hentati[1], Raeid M. M. Abed[2], Nasser Abotalib[1], Ashraf M. El Nayal[1], Ijaz Ashraf[3], Wael Ismail[1,*]

[1]Environmental Biotechnology Program, Life Sciences Department, College of Graduate Studies, Arabian Gulf University, Manama, Bahrain, [2]Biology Department, College of Science, Sultan Qaboos University, Muscat, Oman, [3]Bahrain Petroleum Company, Manama, Bahrain

***CORRESPONDENCE**

Wael Ismail

waelame@agu.edu.bh

Materials and Methods

Table S1. Physicochemical properties of the oily sludge.

Parameters	Value	Unit	Method
pH	5.6	-	BLM_015
Water content	49.63	(wt%)	ASTM_E1064
Kinematic Viscosity (at 50 °C)	273.7	Centistock	ASTM_D7042
TPH	530	g/kg	Tahhan et al. (2011)
Nitrogen (N_2)	681	mg/kg	D-4629
Phosphate	149	mg/kg	BLM_059
Sulfur	1.4	%	ASTM_D4294
Sodium (Na)	14298	mg/kg	ASTM_D1318
Calcium (Ca)	2614	mg/kg	BLM_040
Magnesium (Mg)	1290	mg/kg	CMM_111
Potassium (K)	629	mg/kg	CMM_111
Iron (Fe)	4462	mg/kg	CMM_111
Silicon (Si)	2062	mg/kg	IP_377
Aluminum (Al)	1019	mg/kg	IP_377
Zinc (Zn)	202	mg/kg	CMM_111
Lead (Pb)	71	mg/kg	CMM_111
Copper (Cu)	67	mg/kg	CMM_111
Manganese (Mn)	28	mg/kg	A_A_S
Vanadium (V)	26	mg/kg	CMM_111
Nickel (Ni)	21	mg/kg	CMM_111

Table S2. Composition of the chemically defined medium (CDM). Components highlighted in yellow constitute the basal medium which is supplemented with all the other components (the complement).

**Component**	**Final Concentration (g/L)**
KH_2PO_4	1.08
K_2HPO_4	5.6
NH_4Cl	0.54
$MgCl_2.6H_2O$	0.2
$CaCl_2.2H_2O$	0.044
$FeCl_2.4H_2O$	1.5
**Vitamins**	
Cyanocobalamin	0.2×10^{-3}
Pyridoxamine-HCl	0.6×10^{-3}
Thiamin-HCl	0.4×10^{-3}
Nicotinic acid	0.4×10^{-3}
p-Aminobenzoate	0.32×10^{-3}
Biotin	0.04×10^{-3}
Ca-pantothenate	0.4×10^{-3}
**Trace elements**	
$ZnCl_2.7H_2O$	70×10^{-6}
$MnCl_2.4H_2O$	100×10^{-6}
$CuCl_2$	20×10^{-6}
$CoCl_2.6H_2O$	200×10^{-6}
$Na_2MoO_4.2H_2O$	40×10^{-6}
$NiCl_2.6H_2O$	20×10^{-6}
H_3BO_3	20×10^{-6}

Workflow for Illumina MiSeq amplicon sequencing and analysis

Total genomic DNA was extracted and DNA concentration and purity were monitored on 1% agarose gels. According to the concentration, DNA was diluted to 1 ng/µL using sterile water. The 16S rRNA V3-V4 hypervariable region was amplified using the primers 341F and 805R with the barcodes. All PCR reactions were carried out in 25 µL with 0.5 µL of KAPA®High-Fidelity PCR Master Mix (KAPA BIOSYSTEMS), 0.5 µM of forward and reverse primers, and about 1 ng template DNA. Thermal cycling started with the initial denaturation at 95℃ for 3 min, followed by 30 cycles of denaturation at 95℃ for 30 sec, annealing at 57 ℃ for 30 sec, elongation at 72 ℃ for 30 sec, and final extension at 72 ℃ for 5 min. For PCR products quantification and qualification, an equal volume of 1 x loading buffer (contained SYB green) was mixed with the PCR products and analyzed by electrophoresis on 2% agarose gel for detection. Samples with one bright main band between 450-500 bp were chosen for further experiments. The PCR products were mixed in equal ratios and purified with QIAquick Gel Extraction kit (QIAGEN). Sequencing libraries were generated using Truseq nano DNA Library Prep kit (Illumina, USA) following the manufacturer's instructions and index codes were added. The library quality was assessed on Qubit 2.0 Fluorometer (Thermo Scientific) and Agilent Bioanalyzer 2100 system. At last, the library was sequenced on an Illumina-MiSeq platform, generating 300 bp paired-end reads.

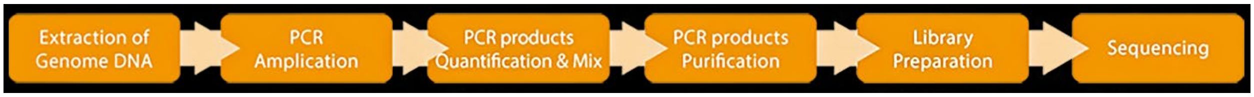

Workflow-information analysis process

The main tool of the workflow was QIIME (Caporaso et al., 2010), which contained multiple analysis process programs.

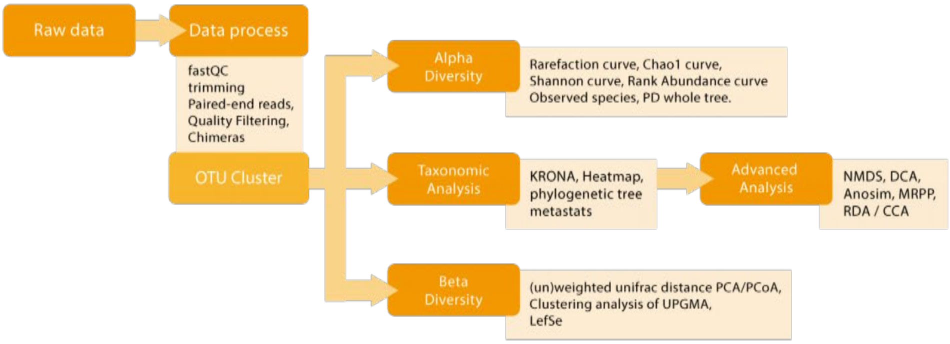

Caporaso JG, KuczynskiJ, StombaughJ, et al. (2010). QIIME allows analysis of high-throughput community sequencing data. Nat Methods, 7(5): 335-336.

Results

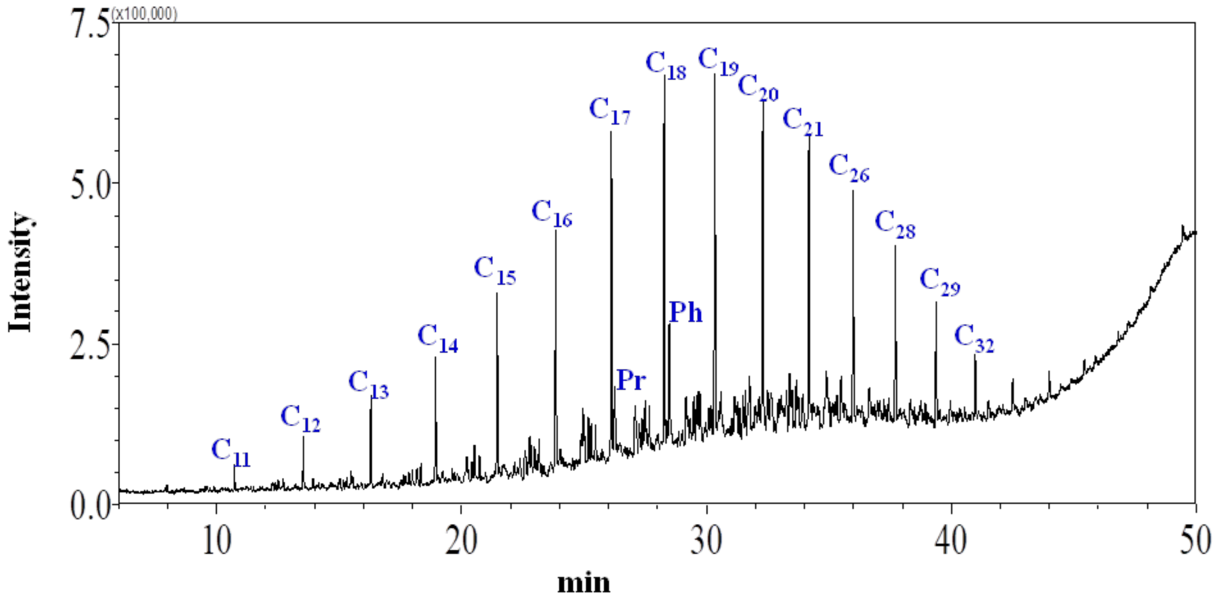

Figure S1. Total ion chromatogram of oily sludge hydrocarbons. The *n*-alkanes distribution is designated as C_{11}-C_{32}. (Pr): pristane; (Ph): phytane.

Table S3. Hydrocarbon components extracted from the oily sludge.

**Compound Name**	**Molecular formula**	**Molecular mass (g/mol)**
**Alkanes**		
Undecane	$C_{11}H_{24}$	156.31
Dodecane	$C_{12}H_{26}$	170.33
Tridecane	$C_{13}H_{28}$	184.37
Tetradecane	$C_{14}H_{30}$	198.39
Pentadecane	$C_{15}H_{32}$	212.42
Hexadecane	$C_{16}H_{34}$	226.41
Heptadecane	$C_{17}H_{36}$	240.48
Ocatdecane	$C_{18}H_{38}$	254.5
Nonadecane	$C_{19}H_{40}$	268.52
Eicosane	$C_{20}H_{42}$	282.54
Heneicosane	$C_{21}H_{44}$	296.58
Hexacosane	$C_{26}H_{54}$	366.71
Octacosane	$C_{28}H_{58}$	394.77
Nonacosane	$C_{29}H_{60}$	408.6
Dotriacontane	$C_{32}H_{66}$	450.9
**Isoprepenoids**		
2,6,10,14-tetramethylpentadecane: Pristane	$C_{19}H_{40}$	268.5
2,6,10,14-tetramethylhexadecane: Phytane	$C_{20}H_{42}$	282.5

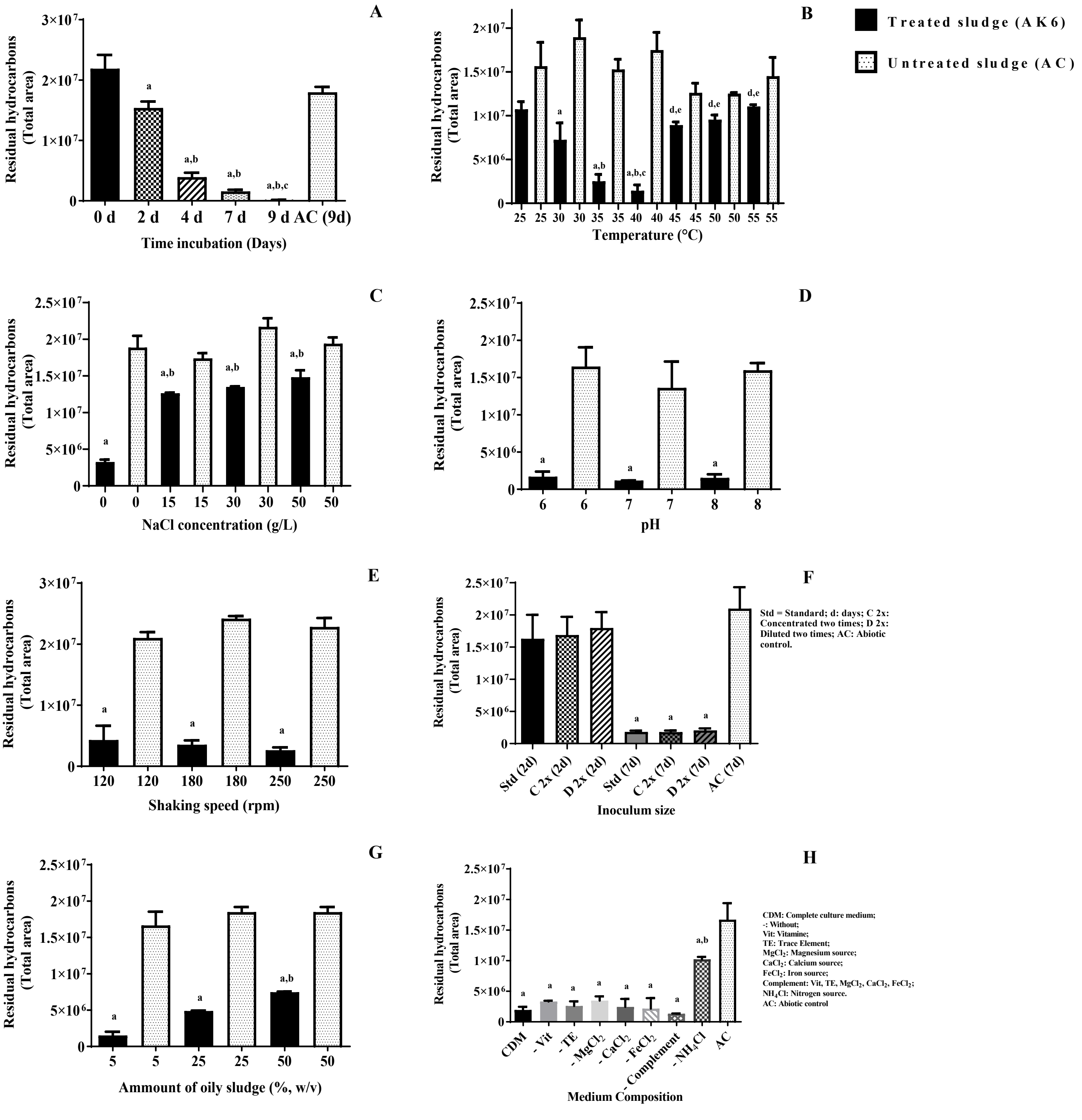

Figure S2. Residual hydrocarbons after treatment of oily sludge by the mixed culture AK6 compared to untreated sludge (Abiotic control, AC) under different bioprocess conditions: incubation time (A); temperature (B); NaCl concentration (C); pH (D); shaking speed (E); inoculum size (F); oily sludge concentration (G) and medium composition (H). Values given represent the mean of two replicates ± standard deviation. vs: versus. [a] $p < 0.05$ treated groups by AK6 vs.

untreated groups (AC); **(A)**: [b] $p < 0.05$ at 2 days vs. other incubation time; [c] $p < 0.05$ at 4 days vs. other incubation times; **(B)**: $p < 0.05$ at 25 °C vs. other temperatures; [c] $p < 0.05$ at 30 °C vs. other temperatures; [d] $p < 0.05$ at 35 °C vs. other temperatures; [e] $p < 0.05$ at 40 °C vs. other temperatures; **(C)** [b] $p < 0.05$: at 0 g/L NaCl vs. salinities; [c] $p < 0.05$ at 15 g/L NaCl vs. other salinities; **(G)** [b] $p < 0.05$: at 5% oily sludge vs. other oily sludge concentrations; **(H)**: [b] $p < 0.05$ in complete culture medium vs. medium without complement (vitamins, trace elements, $MgCl_2$, $CaCl_2$, $FeCl_2$) and medium without NH_4Cl. The pH, shaking speed and inoculum size had no significant influence ($p > 0.05$) on hydrocarbon removal by AK6.
